# Spontaneous tumor lysis syndrome following liver biopsy: a case report and literature review

**DOI:** 10.3389/fonc.2025.1683025

**Published:** 2025-11-12

**Authors:** Wenchao Mao, Xiangyang Jiang, Minjia Wang, Kailun Cai, Weihang Hu, Shijin Gong, Yuexi Zhao

**Affiliations:** 1 Department of Critical Care Medicine, Zhejiang Hospital, Hangzhou, China; 2 Department of Endocrinology, Zhejiang Hospital, Hangzhou, China

**Keywords:** tumor lysis syndrome, spontaneous tumor lysis syndrome, liver biopsy, solid tumors, case report, continuous renal replacement therapy

## Abstract

**Background:**

Tumor lysis syndrome (TLS), characterized by electrolyte imbalances and acute kidney injury, predominantly occurs following cytotoxic chemotherapy in hematologic malignancies. Spontaneous TLS (STLS) in solid tumors remains rare. This report describes STLS induced by a diagnostic liver biopsy and reviews the literature on procedure-associated TLS.

**Case presentation:**

An 84-year-old male presented with extensive hepatic metastases and markedly elevated tumor markers. Ultrasound-guided percutaneous liver biopsy confirmed the diagnosis of metastatic adenocarcinoma. Within 24 hours post-procedure, the patient developed acute respiratory failure, anuria, severe metabolic acidosis (pH 7.23), hyperkalemia (5.5 mmol/L), acute kidney injury (creatinine 299 μmol/L), hyperuricemia (716 μmol/L), and elevated lactate dehydrogenase (3953 U/L), fulfilling the diagnostic criteria for TLS. Concurrent hemothorax occurred. Continuous renal replacement therapy (CRRT) achieved rapid correction of metabolic derangements, with parameters returning to normal within seven days.

**Conclusion:**

Diagnostic liver biopsy can induce STLS in patients with high-burden solid tumors. Our systematic analysis reveals that minimally invasive procedures may precipitate TLS, emphasizing the importance of prophylactic measures, early recognition, and immediate CRRT initiation for optimal outcomes.

## 1 Introduction

Tumor lysis syndrome (TLS) is an oncological emergency characterized by the rapid lysis of malignant cells, resulting in severe metabolic disturbances including hyperkalemia, hyperphosphatemia, hyperuricemia, and hypocalcemia. These biochemical abnormalities can lead to serious complications such as acute kidney injury (AKI), cardiac arrhythmias, and seizures, potentially resulting in death if not promptly managed (1, 2). TLS is predominantly associated with hematologic malignancies following cytotoxic chemotherapy, particularly in rapidly proliferating, high-burden malignancies such as Burkitt lymphoma and acute lymphoblastic leukemia (3). However, spontaneous TLS (STLS) can occur in the absence of antineoplastic treatment, although this phenomenon remains rare (4, 5). In solid tumors, particularly those with hepatic metastases, STLS occurs less frequently. While chemotherapy-related TLS results from massive synchronous cellular lysis following therapeutic intervention, STLS may be precipitated by mechanical disruption during diagnostic procedures, tumor necrosis, or spontaneous cellular lysis in high-burden disease states (6). One case report described a pediatric patient who developed hyperthermia, cardiac arrhythmias, severe hyperkalemia, and metabolic acidosis during the biopsy incision closure, demonstrating the potential for invasive procedures to precipitate STLS (6).

Current knowledge regarding procedure-induced STLS remains limited, with only scattered case reports in the literature and an absence of standardized prevention and treatment protocols. Here, we report a case of an 84-year-old male with extensive hepatic metastases who developed STLS following a percutaneous liver biopsy. Through detailed analysis of this case and a systematic review of analogous cases, we aim to enhance clinical awareness of this rare but potentially lethal complication and emphasize the critical importance of vigilant monitoring and prophylactic management in high-risk patients.

## 2 Case report

### 2.1 Patient information

**Figure 1:**
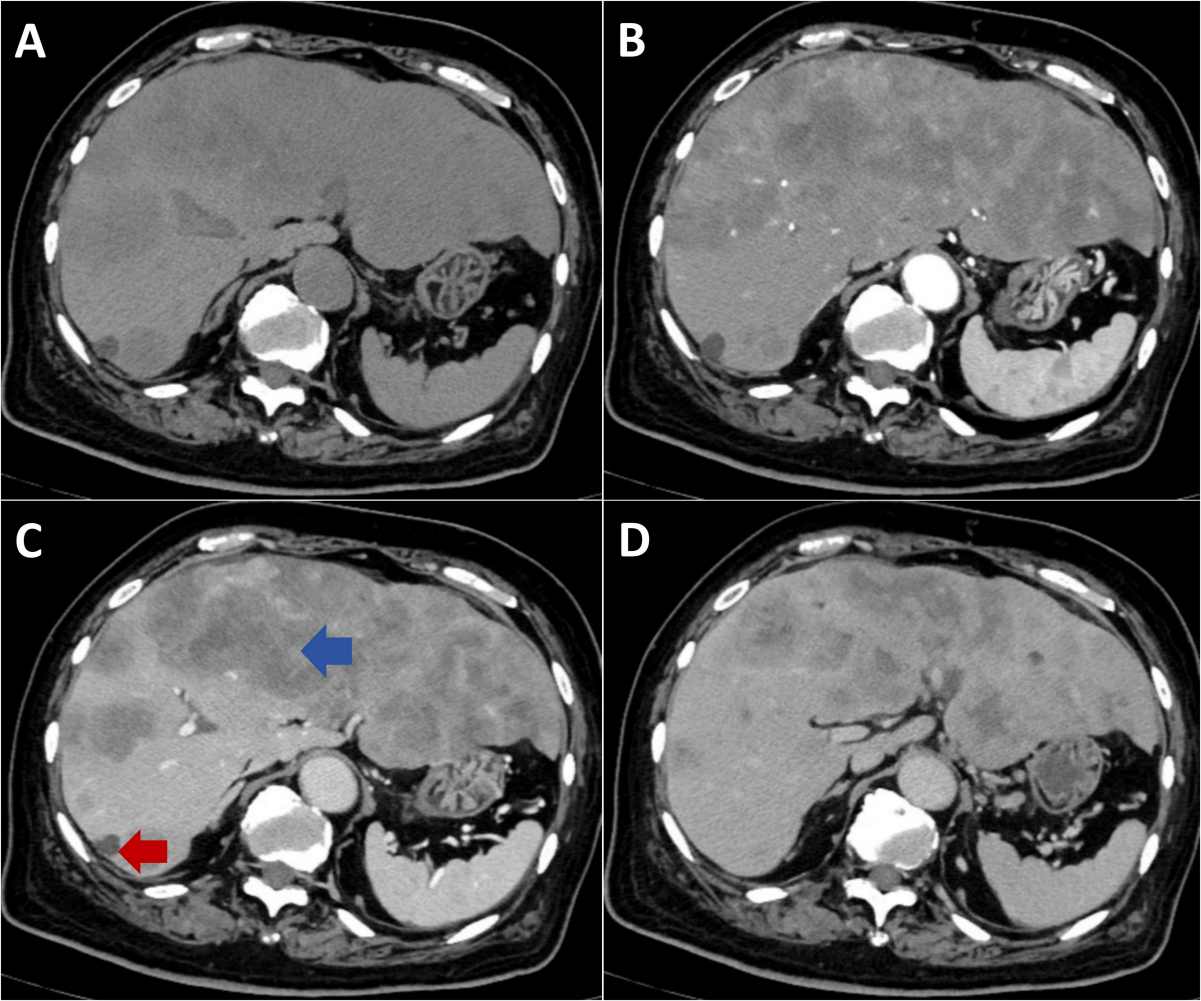
Contrast-enhanced CT imaging of metastatic hepatic lesions. **(A)** Non-contrast phase shows multiple hypodense lesions with ill-defined margins and coalescence throughout the liver. **(B)** Arterial phase demonstrates irregular peripheral rim enhancement with central hypoattenuation. **(C)** Portal venous phase reveals progressive peripheral enhancement. **(D)** Delayed phase shows sustained rim enhancement with minimal central fill-in.

An 84-year-old male presented with a one-month history of fatigue and altered mental status. His medical history included urothelial carcinoma diagnosed 10 years previously, treated with radical cystectomy and urinary diversion. A detailed family history was obtained, which revealed no instances of gastrointestinal or other malignant tumors. Contrast-enhanced abdominal computed tomography revealed multiple hepatic hypodense lesions, with the largest measuring 6.2×3.5 cm (**Figure 1**, blue arrow), and showing ill-defined margins and partial confluence. Contrast-enhanced imaging demonstrated irregular rim enhancement (**Figure 1**). The gastric antrum showed wall thickening with preserved mucosal continuity (**Supplementary Figure 1**). Multiple perigastric and retroperitoneal lymph nodes demonstrated mild enhancement (**Supplementary Figures 2**, **3**).

Serum biochemical parameters on admission showed the following values: alanine aminotransferase 22 U/L, aspartate aminotransferase 190 U/L, lactate dehydrogenase 1404 U/L, creatine kinase 392 U/L, and uric acid 529 μmol/L (**Table 1**). Electrolyte concentrations and creatinine were within normal limits. Tumor markers were significantly elevated: carcinoembryonic antigen 8970 ng/mL, carbohydrate antigen 125 255.6 U/mL, carbohydrate antigen 19-9 >10000 U/mL, carbohydrate antigen 72-4 >250 U/mL, neuron-specific enolase 61.17 ng/mL, cytokeratin 19 fragment 21-1 541.4 ng/mL, carbohydrate antigen 242 >200 IU/mL, and carbohydrate antigen 50 >500 IU/mL. Baseline inflammatory markers were mildly elevated, with a white blood cell (WBC) count of 9.14×10^9^/L, neutrophils at 64.7%, a C-reactive protein (CRP) level of 39.9 mg/L, and a procalcitonin level of 0.30 ng/mL (**Table 1**). There was no clinical evidence of fever or infection.

**Table 1:** Hemodynamic and laboratory measurements during hospitalization.

Time after admission	Day 0 Admission	Day 2 Before Liver Biopsy	Day 3 ICU Admission	Day 4 Under-CRRT	Day 8 CRRT Weaning
Hemodynamic Parameters					
Blood pressure (mmHg)	138/82	132/76	143/79	141/59	152/64
Heart rate (beats/min)	81	76	119	86	84
Respiratory rate (breaths/min)	19	19	28	23	16
SpO_2_ (%)	100	100	100	100	100
Temperature (°C)	37.2	36.4	36.7	36.9	36.7
Fluid balance (mL)	–	–	+810	+323	-711
Norepinephrine (µg/kg/min)	–	–	0.06	0.02	–
Laboratory Parameters					
pH	–	7.42	7.23	7.40	7.44
PaO2 (mmHg)	–	98.2	72.5	104	121
Base excess (mmol/L)	–	-3.3	-16.8	-3.6	1.6
Lactate (mmol/L)	1.90	1.70	11.70	5.50	2.10
Potassium (mmol/L)	3.84	4.0	5.5	4.5	4.12
Phosphorus (mmol/L)	0.84	0.81	2.49	1.62	0.91
Calcium (mmol/L)	2.17	2.19	1.92	2.09	2.33
Uric acid (μmol/L)	529	533	716	446	176
LDH (U/L)	1404	1436	3953	2366	1521
Creatinine (μmol/L)	109	112	299	217	166
Hemoglobin (g/L)	139	141	125	117	106
White blood cell (x10^∧^9/L)	9.14	9.33	13.06	13.9	8.87
Neutrophil percentage (%)	64.7	68.2	81.6	80.3	76.1
CRP (mg/L)	39.88	42.36	76.87	106.57	41.22
Procalcitonin (ng/mL)	–	0.30	0.42	0.37	–
Blood culture	–	–	Negative	–	–

CRRT, continuous renal replacement therapy; CRP, C-reactive protein; ICU, intensive care unit; LDH, lactate dehydrogenase; PaO₂, partial pressure of oxygen; SpO₂, oxygen saturation. Ellipses (-) indicate data not available.

### 2.2 Diagnostic and therapeutic process

**Figure 2:**
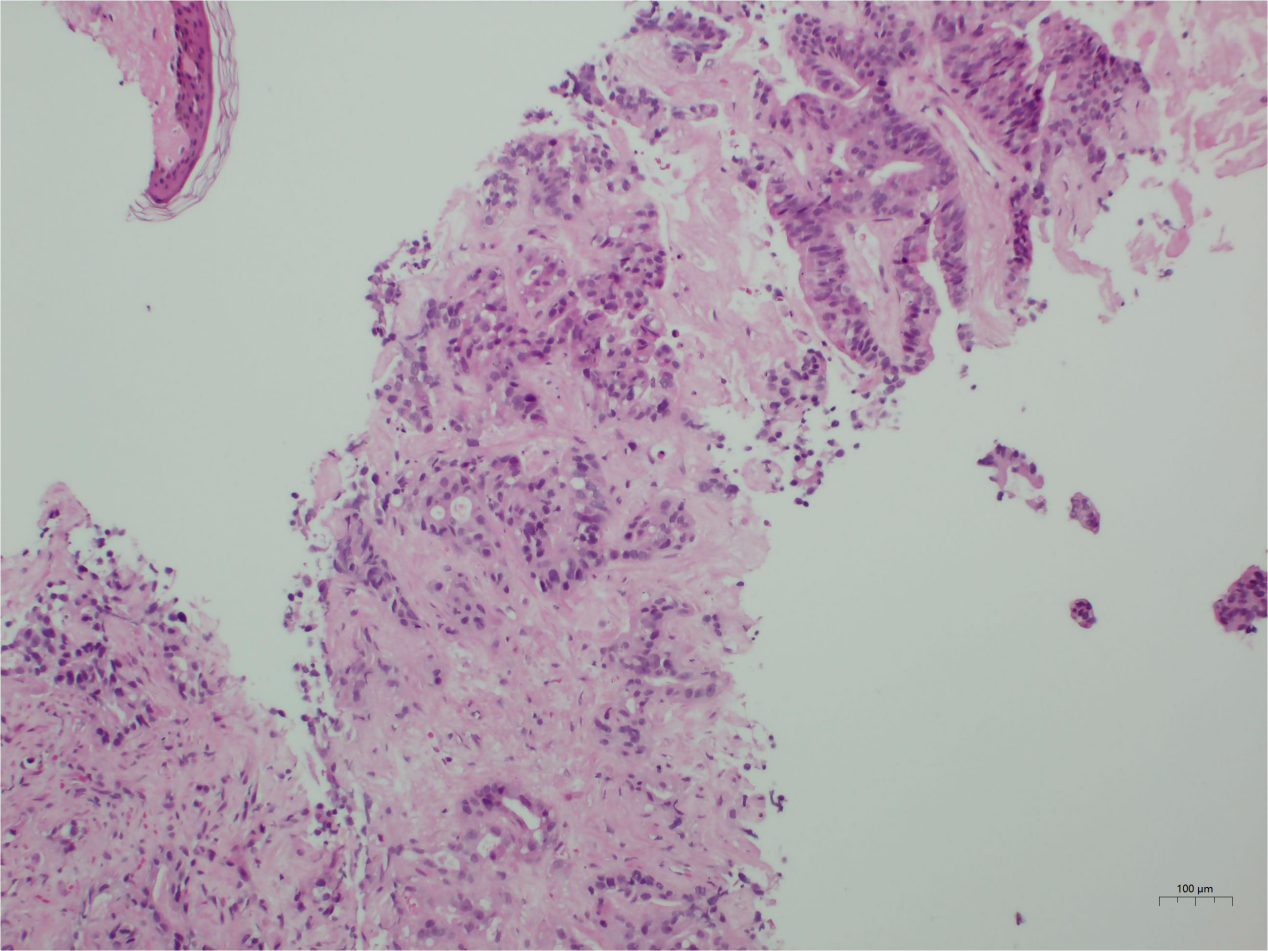
Histological findings. The tumor is identified as a moderately differentiated adenocarcinoma. Immunohistochemical profiling indicates a likely gastrointestinal origin.

Given the markedly elevated tumor markers and extensive hepatic involvement, an ultrasound-guided percutaneous liver biopsy was performed on the second day of admission to establish a prompt histological diagnosis. Under real-time ultrasonographic guidance, an 18-gauge automatic core biopsy needle was inserted through the right midclavicular line, 4 cm below the nipple, following local infiltration anesthesia with 1% lidocaine. Tissue specimens were obtained for histopathological examination. Liver biopsy revealed a moderately differentiated adenocarcinoma (**Figure 2**). Based on the immunohistochemical profile (CDX2 positive, SATB2 focal positive, CK19 positive, CK7 partial positive, HepPar-1 positive, AFP negative), the overall features favor a metastatic adenocarcinoma of gastrointestinal origin. However, the primary site of the tumor remained presumptive, as a gastrointestinal endoscopic biopsy was not performed due to the family’s subsequent refusal of esophagogastroduodenoscopy (EGD) and colonoscopy.

**Figure 3:**
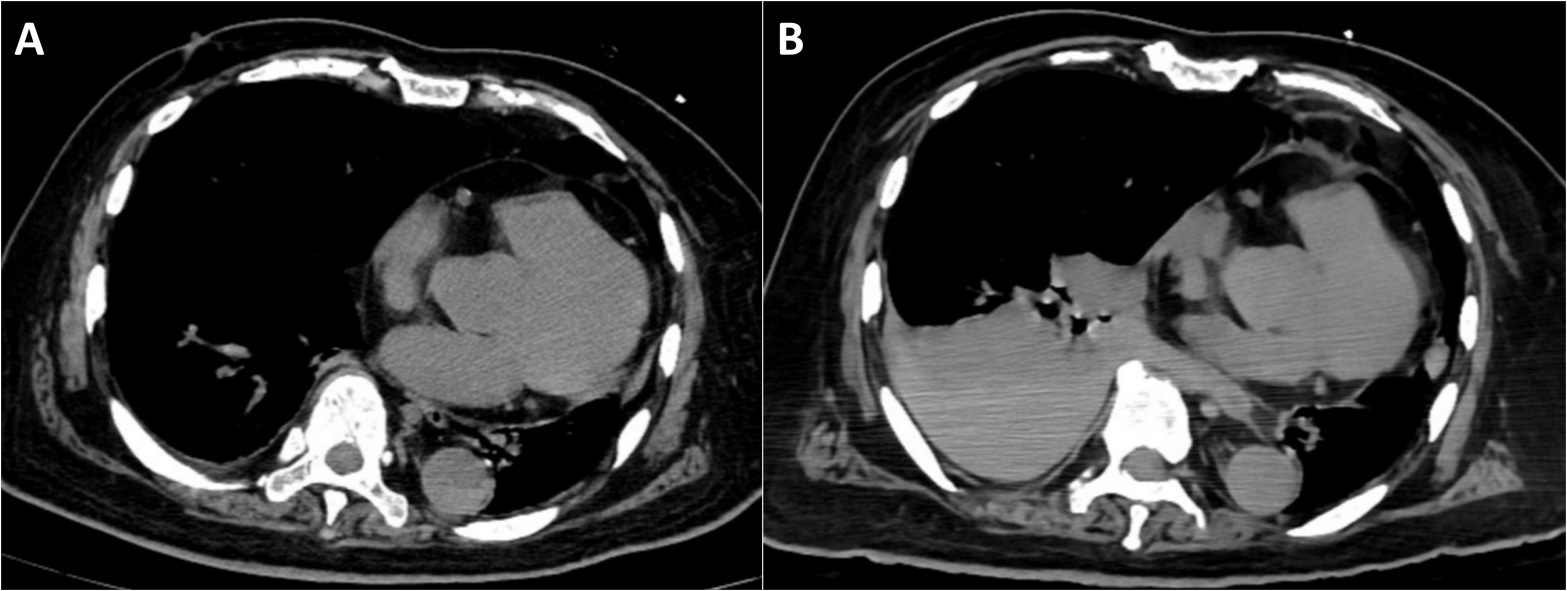
Chest computed tomography. **(A)** CT on the day of admission shows no significant pleural effusion. **(B)** Right-sided pleural effusion (possible hemothorax).

Following the liver biopsy, the patient experienced significant right upper quadrant pain, which was refractory to tramadol and morphine administration. On the second post-procedural day, he developed an acute onset of chest tightness, dyspnea, altered consciousness, hypoxemia, and 6-hour anuria. Laboratory investigations revealed: arterial pH 7.23, partial pressure of carbon dioxide 20.5 mmHg, partial pressure of oxygen 72.5 mmHg, base excess -16.8 mmol/L, serum potassium 5.5 mmol/L, serum lactate 11.7 mmol/L, alanine aminotransferase 58 U/L, aspartate aminotransferase 1031 U/L, lactate dehydrogenase 3953U/L, serum creatinine 299 μmol/L, serum phosphorus 2.49 mmol/L, and serum calcium 1.92 mmol/L. Initial chest CT on admission (**Figure 3a**) revealed no pleural effusion, while subsequent imaging (**Figure 3b**) demonstrated a new right-sided pleural effusion.

This clinical presentation was consistent with acute tumor lysis syndrome. Although the patient’s WBC count and CRP rose after biopsy (WBC 13.06×10^9/L, neutrophils 81.6%, CRP 76.9 mg/L on Day 3), his procalcitonin remained low (≤0.42 ng/mL) and blood cultures were negative. There was no other source of infection identified. These findings support a noninfectious inflammatory response (tumor cell breakdown) rather than sepsis. Hemodynamically, despite a drop in hemoglobin from a preoperative level of 141 g/L to a postoperative level of 125 g/L, indicative of blood loss, and the presence of shock, the patient only required a transient, low-dose norepinephrine infusion (up to 0.06μg/kg/min) to maintain blood pressure (**Table 1**). In contrast, the patient developed severe metabolic derangements—namely, severe lactic acidosis, hyperkalemia, and AKI—that were disproportionate to the extent of blood loss and are hallmark features of TLS.

With a high clinical suspicion of TLS, the patient was transferred to the intensive care unit (ICU) for aggressive fluid resuscitation and correction of metabolic acidosis. Despite these interventions, the serum lactate concentration continued to rise progressively, metabolic acidosis recurred, and the hyperkalemia remained refractory to conventional therapies. Consequently, continuous renal replacement therapy (CRRT) was promptly initiated, utilizing regional anticoagulation with heparin and protamine sulfate (blood flow rate 180 mL/min, replacement fluid rate 2000 mL/h, predilution 70%, postdilution 30%). Two hours of continuous venovenous hemofiltration effectively corrected hyperkalemia and metabolic acidosis.

Ongoing dyspnea warranted repeat imaging and a multidisciplinary consultation involving radiology, thoracic surgery, oncology, and the department of interventional ultrasound. The hemothorax was attributed to tumor necrosis and hemorrhage from a segment 7 hepatic tumor with diaphragmatic invasion (red arrow, **Figure 1**), excluding procedure-related complications. Therapeutic thoracentesis drained 1000 mL of hemorrhagic pleural fluid, with subsequent resolution of respiratory symptoms.

### 2.3 Outcome

**Figure 4:**
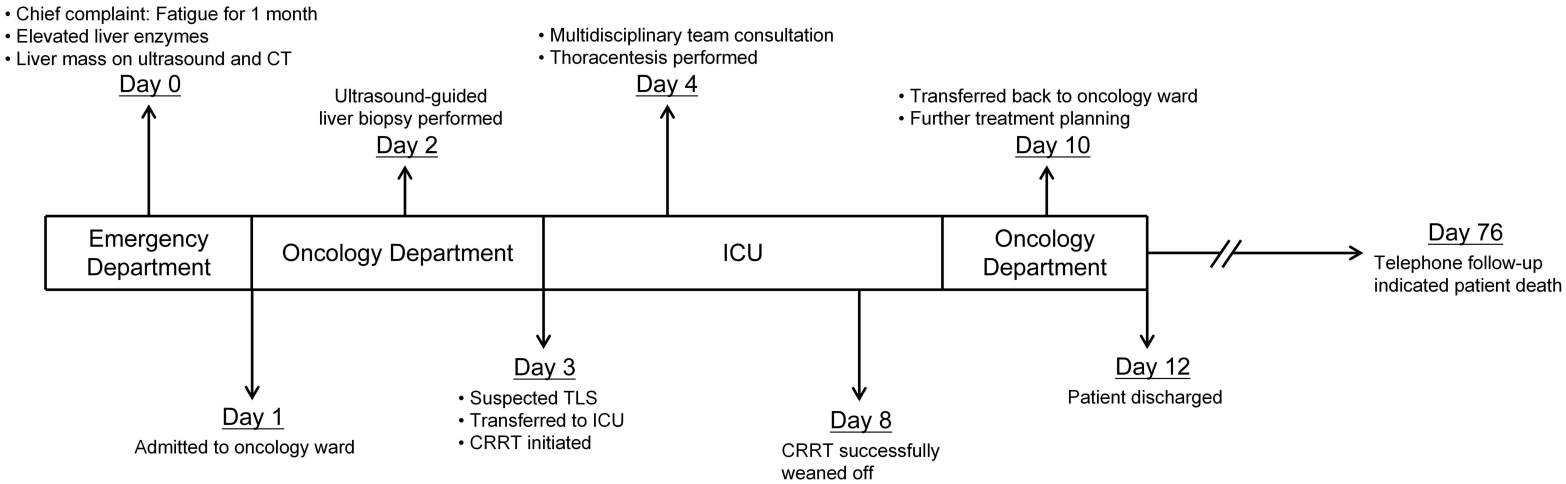
The clinical timeline of the patient.

After receiving CRRT, hepatoprotective agents, and other supportive measures, the patient’s electrolyte disturbances and internal environment were corrected. Liver function gradually improved, and laboratory parameters stabilized (alanine aminotransferase 31 U/L, aspartate aminotransferase 191 U/L, LDH 1484 U/L, alkaline phosphatase 225 U/L, gamma-glutamyl transferase 100 U/L). Renal function returned to normal within the subsequent week. The patient was then transferred to the Department of Oncology for further management. Unfortunately, citing advanced age, financial constraints, and multiple comorbidities, the patient and his family declined further diagnostic workup, including EGD and colonoscopy, to identify the primary tumor site. They also refused any anti-tumor interventions, such as surgery, radiotherapy, or chemotherapy. The patient was discharged on hospital day 12. Subsequent telephone follow-up revealed that the patient expired approximately 2 months after discharge (**Figure 4**).

## 3 Discussion

We report a rare case of TLS occurring after percutaneous liver biopsy in an 84-year-old male with hepatic metastases. The patient developed classic TLS manifestations, including hyperkalemia, hyperuricemia, hyperphosphatemia, AKI, and severe metabolic acidosis, with concurrent hemothorax secondary to diaphragmatic tumor invasion. To better understand this rare complication, we conducted a systematic literature review and identified seven additional cases of procedure-induced TLS, bringing the total cohort to eight cases. The clinical characteristics of these patients are presented in **Table 2**. This comprehensive analysis yields three critical clinical insights (1): diagnostic procedures may precipitate TLS in solid tumors with high tumor burden through identifiable risk factors (2); prophylactic intervention, early recognition, and prompt CRRT are essential for favorable outcomes; and (3) multidisciplinary management is imperative for complex cases.

**Table 2:** Clinical characteristics of eight patients with procedure-induced tumor lysis syndrome.

Author	Gender	Age	Tumor type	Precipitating factor	Tumor size/characteristics	CRRT	Outcome
Our case	Male	84 years	Hepatic metastases	Liver biopsy	Multiple hepatic lesions, largest 6.2×3.5 cm	Yes	Survival
Thomas H. Shin (18)	Male	61 years	Lung cancer	Lobectomy	5.3×4.2×4.2cm	No	Survival
Ho Hung Cheung (19)	Male	64 years	Hepatocellular carcinoma	ALPPS stage I surgery	Right liver 17.3 cm	Yes	Survival
Ankur Verma (20)	Male	40 years	Burkitt lymphoma	Open biopsy + bowel obstruction relief surgery	Extensive abdominopelvic, hepatic, and renal involvement	Yes	Survival
Daniel Pindak (7)	Male	19 years	Retroperitoneal teratoma	Radical resection	33×25×13 cm, multi-organ infiltration	No	Death
M. H. Lee (9)	Male	6 years	Burkitt lymphoma	Exploratory laparotomy	Extensive abdominopelvic involvement	Yes	Survival
Sirui Pan (6)	Male	2 years	Burkitt lymphoma	Open tumor biopsy	Multi-organ involvement (liver, intestine, kidney, peritoneum)	Yes	Survival
Mridul Dhar (8)	Male	5 years	Wilms’ tumor	Giant Wilms’ tumor resection	Right kidney 30×20 cm	No	Death

### 3.1 Diagnostic procedures may precipitate TLS in solid tumors: risk factors and underlying mechanisms

Historically, TLS has been predominantly associated with chemotherapy-induced cytotoxicity in hematologic malignancies, with sporadic occurrence in solid tumors. However, Howard et al. demonstrated that with advances in anticancer therapeutics, the clinical spectrum of TLS is expanding, with an increasing incidence in solid tumor patients due to more aggressive treatment regimens and improved diagnostic recognition (1). Our systematic analysis of eight procedure-induced TLS cases reveals distinct high-risk characteristics that warrant clinical attention.

High tumor burden emerged as the most critical risk factor, present in all cases (8/8). This was characterized by a tumor diameter exceeding 15 cm, multi-organ involvement, or involvement of greater than 50% of the affected organ volume. Our patient exhibited extensive hepatic metastases (maximum diameter 6.2×3.5 cm) with markedly elevated tumor markers (carcinoembryonic antigen 8, 970 ng/mL, carbohydrate antigen 19-9 >10, 000 U/mL). Comparable tumor burden characteristics were documented in similar cases: a 33×25×13 cm teratoma reported by Pindak et al. (7), a 30×20 cm Wilms tumor by Dhar et al. (8), and extensive lymphomatous involvement by Pan et al. (6). Notably, hematological malignancies comprised 42.9% of cases (3/7), with Burkitt lymphoma demonstrating the highest risk profile, while even histologically benign solid tumors such as Wilms tumor conferred substantial TLS risk when associated with an extensive tumor burden.

Baseline laboratory abnormalities constituted additional high-risk indicators, observed in 87.5% of cases (7/8). Our patient’s preoperative lactate dehydrogenase (LDH) elevation (1404 U/L) was comparable to similar cases: 1545 U/L reported by Lee et al. (9) and 1698 U/L by Pan et al. (6). Comprehensive reviews by Howard et al. and Barbar et al. have established LDH as a biomarker of accelerated tumor cell proliferation and tissue destruction, functioning as an independent risk factor for TLS development (1, 10). Other baseline abnormalities included hyperuricemia and renal impairment.

Demographic patterns revealed specific vulnerabilities. The age distribution exhibited a bimodal pattern, with increased risk observed in pediatric patients (≤6 years) and older adults (≥40 years). Remarkably, all affected patients were male, though the significance of this gender predilection requires further investigation.

The pathophysiology underlying procedure-induced TLS involves rapid, massive tumor cell lysis with the subsequent release of intracellular constituents: potassium, phosphate, nucleic acids, and other metabolites (2, 11). Cellular membrane disruption liberates these intracellular components, overwhelming renal clearance mechanisms and precipitating AKI with potentially fatal metabolic derangements (1, 2). Any intervention involving tumor tissue may serve as a precipitating factor through mechanical stress or inflammatory cascade activation. Current guidelines establish a significant TLS risk when the tumor cell mass exceeds 500 g or 300g/m² in pediatric patients, even with minimally invasive procedures (1, 12), which is concordant with our clinical findings.

### 3.2 Critical role of preventive interventions and early CRRT treatment

The temporal onset of procedure-induced TLS demonstrated a characteristic pattern in our analysis: 62.5% of cases manifested intraoperatively (5/8), while 37.5% developed within 24 hours postoperatively (3/8). This temporal clustering strongly supports a direct causal relationship between invasive procedures and TLS development.

Hyperkalemia constituted the predominant biochemical abnormality (8/8), with peak serum potassium concentrations of 5.4-8.3 mmol/L. Additional metabolic derangements included hypocalcemia (7/8, 87.5%), hyperphosphatemia (6/8, 75%), and hyperuricemia (7/8, 87.5%). Life-threatening cardiac arrhythmias were observed in 87.5% of cases (7/8), with cardiac arrest occurring in 37.5% (3/8). Pathognomonic electrocardiographic findings included QRS complex widening, peaked T waves, and a sine wave morphology.

The optimal management approach for TLS prioritizes prevention over therapeutic intervention following syndrome onset. However, our analysis revealed critically inadequate prophylactic measures across all cases. Only two patients received prophylactic hydration (6, 9), administered at suboptimal dosages (<2000 mL/m²/day) without concurrent rasburicase therapy. The remaining cases underwent neither TLS risk stratification nor prophylactic intervention. This absence of preventive measures likely contributed significantly to the adverse clinical outcomes.

Evidence-based guidelines established by Coiffier et al. (12) recommend preventive interventions for high-risk patients, including aggressive hydration (2–3 L/m²/day), uric acid-lowering therapy, and intensive monitoring. The established preventive protocol should encompass: (1) aggressive intravenous hydration initiated 24–48 hours preoperatively, maintaining a urine output >100mL/m²/h; (2) prophylactic uric acid reduction using allopurinol 300mg/day or febuxostat; (3) prophylactic rasburicase administration in high-risk patients; (4) intensive perioperative electrolyte surveillance.

CRRT emerged as a decisive prognostic determinant in our analysis. Patients receiving CRRT achieved 100% survival (5/5), whereas those without effective CRRT experienced 66.7% mortality (2/3). This strong association demonstrates that CRRT represents a critical therapeutic intervention for successful TLS management.

In our case, hyperkalemia and metabolic acidosis were corrected within 2 hours of CRRT initiation, with complete renal function recovery within one week, consistent with outcomes reported by Pan et al. (6). Of particular clinical significance, Dhar et al. (8) identified hyperkalemia and attempted intermittent hemodialysis; however, therapy could not be sustained due to hemodynamic instability, resulting in treatment failure. This observation underscores the therapeutic advantages of CRRT over conventional intermittent hemodialysis, attributed to its continuous slow-efficiency treatment, precise volume management, and minimal hemodynamic perturbation (13, 14). Wilson et al. (15) demonstrated that CRRT provides sustained solute clearance with hemodynamic stability, making it the preferred modality for hemodynamically compromised TLS patients.

### 3.3 Importance of multidisciplinary collaboration in complex cases

TLS, a multi-system metabolic emergency, necessitates multidisciplinary collaboration to achieve optimal clinical outcomes. Such collaboration significantly enhances diagnostic precision and therapeutic decision-making, particularly in complex or high-risk patient populations (16). The successful management of our case, along with the literature analysis, emphasizes several key aspects of multidisciplinary care.

The Journal of Clinical Oncology guidelines emphasize that TLS prevention and management require standardized multidisciplinary care pathways (12). The recommended framework encompasses (1): identification and risk stratification of high-risk patients; (2) development of standardized prophylactic and therapeutic protocols; (3) establishment of round-the-clock nephrology consultation services; (4) provision of CRRT capabilities and specialized personnel; and (5) implementation of comprehensive patient education and surveillance programs.

The hemothorax complication in our case significantly increased the clinical complexity. A multidisciplinary consultation involving radiology, thoracic surgery, oncology, and the department of interventional ultrasound established that the hemothorax resulted from diaphragmatic tumor invasion with hemorrhage secondary to tumor necrosis, rather than procedure-related complications. This precise diagnostic assessment demonstrates the value of multidisciplinary collaboration. Without multidisciplinary input, a misdiagnosis as a procedure-related hemothorax would likely have occurred, compromising subsequent therapeutic strategies.

A recent multicenter study confirmed the importance of multidisciplinary team management. Under standardized collaborative protocols, TLS-related mortality was significantly reduced, and hospital length of stay was markedly decreased (17). These findings provide robust evidence supporting standardized TLS care delivery systems.

Multidisciplinary collaboration should incorporate systematic quality improvement initiatives. Howard et al. (1) advocate for regular TLS case reviews, comprehensive outcome analysis, and continuous care pathway optimization. Based on our analysis of eight cases, we recommend the development of standardized risk assessment tools specifically for procedure-induced TLS in solid tumors, incorporating tumor burden metrics, baseline laboratory parameters, and patient demographics.

## 4 Conclusion

This case demonstrates that patients with high tumor burden may develop TLS following diagnostic interventions, underscoring the importance of prophylactic measures and early CRRT initiation and confirming the critical role of multidisciplinary care coordination. These observations provide significant clinical insights for enhancing TLS prevention strategies and patient outcomes. However, our analysis encompasses only eight cases, potentially introducing selection bias. Larger prospective studies are required to validate these findings.

## Data Availability

The original contributions presented in the study are included in the article/**Supplementary Material**. Further inquiries can be directed to the corresponding author.

## Supplementary material

Supplementary Figure 1 Contrast-enhanced CT imaging of gastric antral thickening. Diffuse wall thickening of the gastric antrum (red arrows) shows mild enhancement on contrast-enhanced imaging with gradual wash-out on delayed phase. **(A)** Non-contrast phase. **(B)** Arterial phase. **(C)** Venous phase. **(D)** Delayed phase.

Supplementary Figure 2 Contrast-enhanced CT imaging of perigastric lymphadenopathy. Enlarged perigastric lymph nodes (red arrows) demonstrate mild enhancement on contrast-enhanced imaging, suggestive of metastatic lymphadenopathy. **(A)** Non-contrast phase. **(B)** Arterial phase. **(C)** Venous phase. **(D)** Delayed phase.

Supplementary Figure 3 Contrast-enhanced CT imaging of retroperitoneal lymphadenopathy. Enlarged retroperitoneal lymph nodes (red arrows) demonstrate mild enhancement on contrast-enhanced imaging, suggestive of metastatic lymphadenopathy. **(A)** Non-contrast phase. **(B)** Arterial phase. **(C)** Venous phase. **(D)** Delayed phase.
