# Supplementary figures and images for: Spontaneous tumor lysis syndrome following liver biopsy: a case report and literature review
Source: Front Oncol. 2025 Nov 12;15:1683025. doi: 10.3389/fonc.2025.1683025 (PMC12646902; doi:10.3389/fonc.2025.1683025)

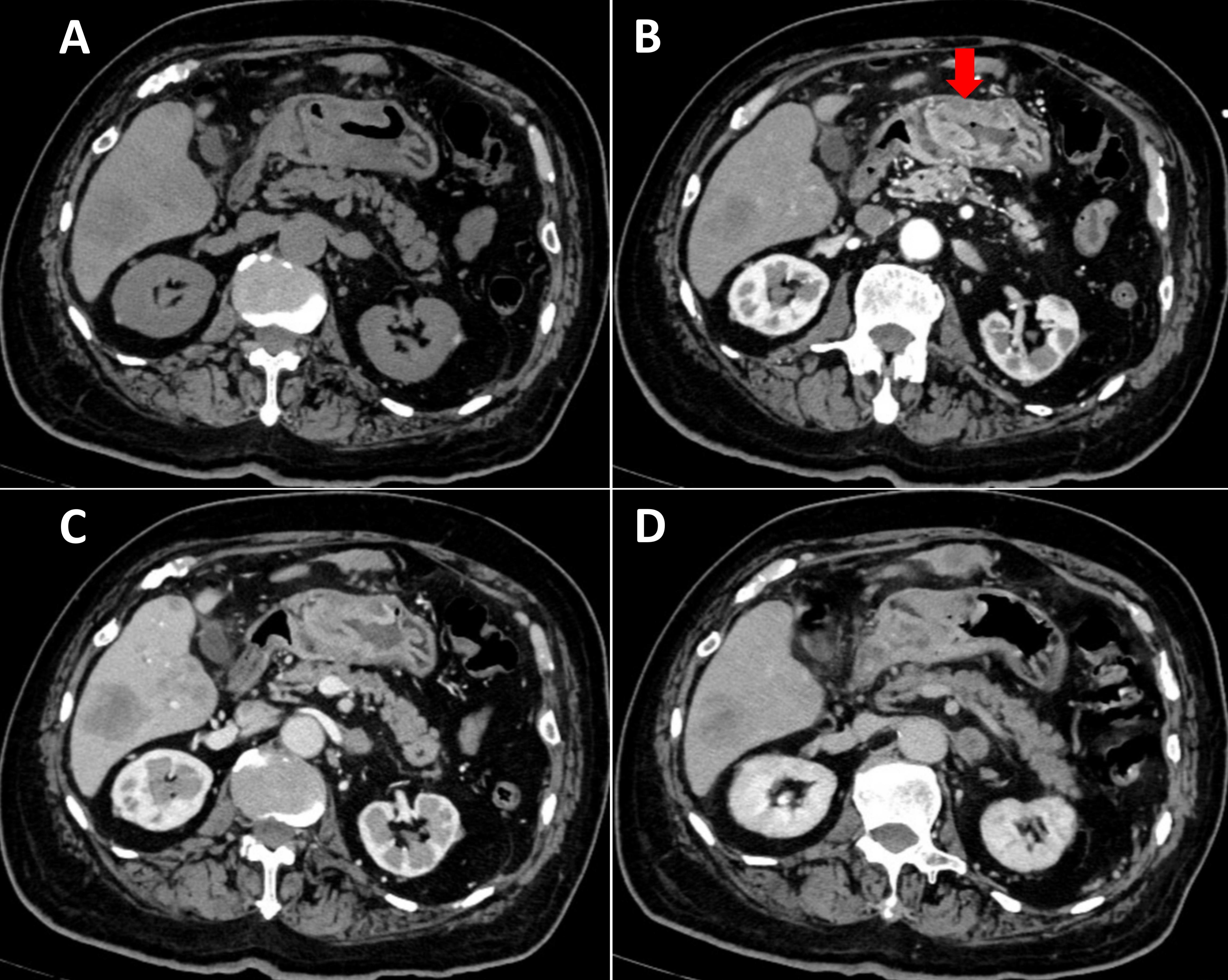

Supplement: Supplementary Figure 1 — Contrast-enhanced CT imaging of gastric antral thickening. Diffuse wall thickening of the gastric antrum (red arrows) shows mild enhancement on contrast-enhanced imaging with gradual wash-out on delayed phase. (A) Non-contrast phase. (B) Arterial phase. (C) Venous phase. (D) Delayed phase. [file Image1.jpeg]

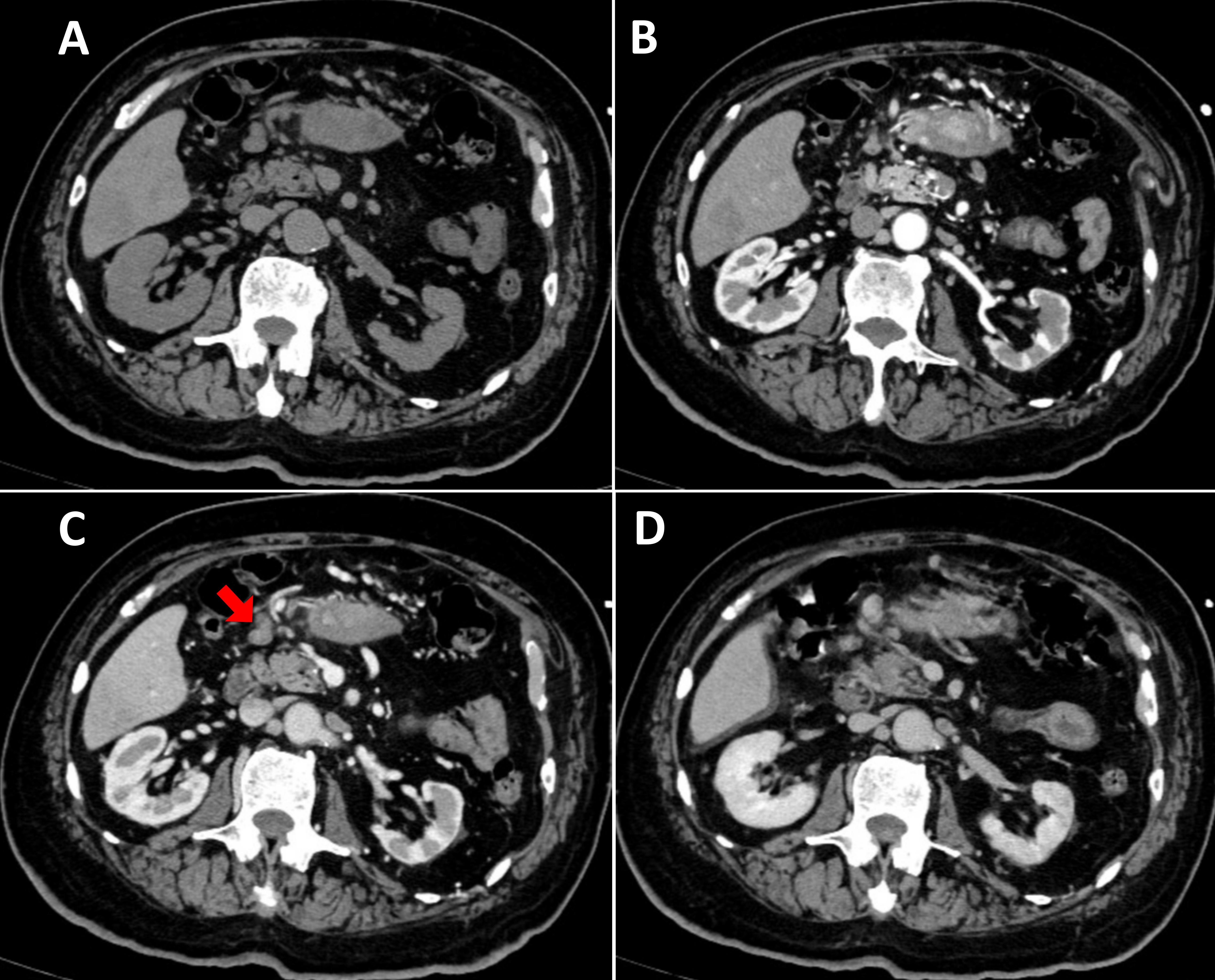

Supplement: Supplementary Figure 2 — Contrast-enhanced CT imaging of perigastric lymphadenopathy. Enlarged perigastric lymph nodes (red arrows) demonstrate mild enhancement on contrast-enhanced imaging, suggestive of metastatic lymphadenopathy. (A) Non-contrast phase. (B) Arterial phase. (C) Venous phase. (D) Delayed phase. [file Image2.jpeg]

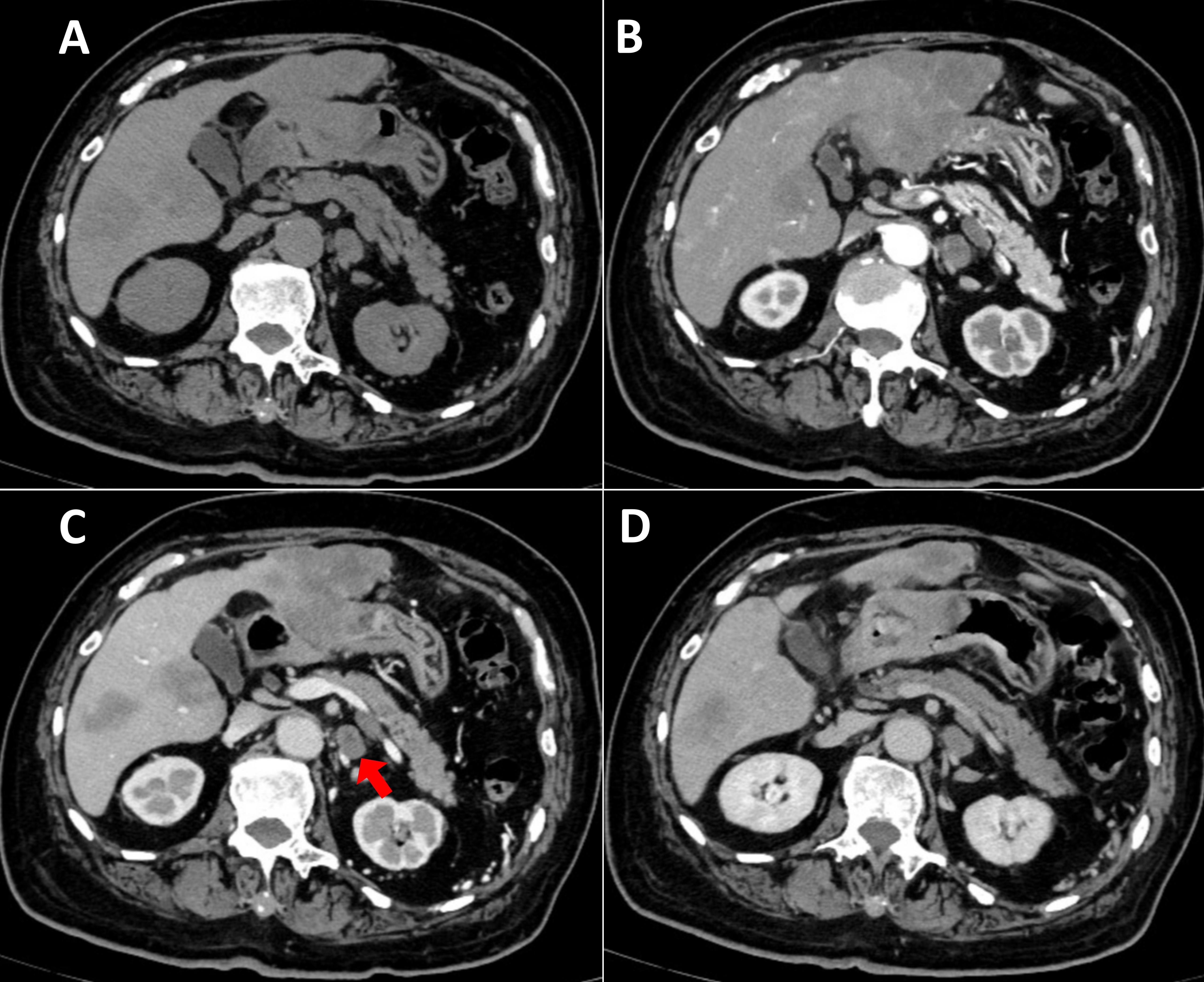

Supplement: Supplementary Figure 3 — Contrast-enhanced CT imaging of retroperitoneal lymphadenopathy. Enlarged retroperitoneal lymph nodes (red arrows) demonstrate mild enhancement on contrast-enhanced imaging, suggestive of metastatic lymphadenopathy. (A) Non-contrast phase. (B) Arterial phase. (C) Venous phase. (D) Delayed phase. [file Image3.jpeg]
